# Identification of LncRNA CARD8-AS1 as a Potential Prognostic Biomarker Associated With Progression of Lung Adenocarcinoma

**DOI:** 10.3389/bjbs.2022.10498

**Published:** 2022-06-23

**Authors:** Yong Ji, Guoqing Zhang, Xingyi Zhang

**Affiliations:** ^1^ Department of Respiratory, Shanghai General Hospital - Songjiang South Campus, Shanghai, China; ^2^ Department of Respiratory, Shanghai General Hospital - Jiading Campus, Shanghai, China

**Keywords:** CARD8-AS1, miR-650, lung adenocarcinoma, proliferation, migration, invasion

## Abstract

**Introduction:** Long non-coding RNAs (lncRNAs) exhibit crucial roles in human tumors. However, the role of lncRNA CARD8-AS1 in lung adenocarcinoma remains elusive. This study investigated the role of CARD8-AS1 in lung adenocarcinoma.

**Materials and Methods:** The expression of CARD8-AS1 was detected by RT-qPCR analysis and confirmed using an online database. The clinical value of CARD8-AS1 was evaluated using the Kaplan-Meier curve and multivariate Cox regression analyses. The effects of CARD8-AS1 on cancer cell proliferation, migration, and invasion potential were assessed through several cellular experiments. Western blot assay was used to measure Bcl-2 and Bax protein levels. The interaction among CARD8-AS1, miR-650, and Bax, was assessed using a dual-luciferase reporter assay.

**Results:** The expression of CARD8-AS1 was decreased in lung adenocarcinoma tissues and cell lines (*p* < 0.001). Low expression of CARD8-AS1 was related to tumor size (*p* = 0.042), TNM stage (*p* = 0.021), lymph node metastasis (*p* = 0.025), and poor overall survival (*p* < 0.05). Elevated expression of CARD8-AS1 could suppress cellular viability, migration potential, and invasion ability (*p* < 0.05). The Bcl-2 protein levels were decreased while Bax levels were increased by overexpression of CARD8-AS1 (*p* < 0.001). miR-650 may thus be a direct target of CARD8-AS1 and Bax may be a direct target of miR-650.

**Discussion:** CARD8-AS1 expression was downregulated in lung adenocarcinoma and associated with several clinical parameters. CARD8-AS1 exerted tumor-suppressive effects by targeting the miR-650 and then regulating Bax expression. CARD8-AS1/miR-650 may serve as novel prognostic biomarkers and potential therapeutic targets for the treatment of lung adenocarcinoma.

## Introduction

Non-small cell lung cancer (NSCLC) is the main histological type of lung cancer (accounts for approximately 85% of all newly diagnosed cases), while lung adenocarcinoma, a sub-classification of NSCLC shows high morbidity and mortality, [1]. In the past decades, considerable progress has been made in surgical treatment, radiotherapy, chemotherapy, molecular targeted therapy, and immunotherapy for lung cancer [2–4]. However, most NSCLC patients are already in the advanced stage during their initial diagnosis, resulting in a poor five-year overall survival rate of these patients [5]. In order to improve survival from lung adenocarcinoma, new biomarkers, therapeutic targets, and drugs for treating patients with lung adenocarcinoma are needed.

Long non-coding RNAs (lncRNAs) have been identified using high-throughput sequencing technologies. They represent a class of non-coding RNAs with lengths of 200 nucleotides in length [6]. Previous studies have demonstrated that lncRNAs play a role in the regulation of cell cycle, and cell differentiation [7]. Accumulating evidence suggests that lncRNAs are aberrantly regulated in various cancer types and may exhibit crucial roles in tumor progression, along with cell migration, proliferation, genomic stability, and survival in patients with lung adenocarcinoma [8–10]. The expression of lncRNAs is involved in major mechanisms of cancer-related gene expression regulation, including transcription regulation [11]. Therefore, the mechanisms underlying molecular regulation of tumor-related lncRNAs in lung adenocarcinoma have become the focus of research [12].

Many lncRNAs are involved in the progression of lung adenocarcinomas, including lncRNA ZFAS1 [13] and lncRNA GMDS-AS1[9]. LncRNA CARD8-AS1 (CARD8-AS1), located on chromosome 19, is reportedly dysregulated in ovarian cancer and glioma and associated with patients’ survival [14, 15]. A recent study has identified several abnormally expressed lncRNAs in lung cancer, including GATA6-AS1, ADAMTS9-AS2, and CARD8-AS1 [16]. Nevertheless, the detailed role of CARD8-AS1 in lung adenocarcinoma remains unclear.

In this study, CARD8-AS1 in lung adenocarcinoma was detected at low levels compared to adjacent non-malignant tissue. The abnormal expression of CARD8-AS1 was significantly related to the clinical outcome of patients with lung adenocarcinoma. The increased expression of CARD8-AS1 could weaken tumor cell proliferative capacities, migrative abilities, and invasion potential. miR-650 may serve as a sponge for CARD8-AS1. These results suggest that CARD8-AS1 plays a crucial role in tumor progression in lung adenocarcinoma and may serve as a potential prognostic marker.

## Materials and Methods

### Collection of Lung Adenocarcinoma Tissue Specimens

The study protocol was approved by the Ethics Committee of Shanghai General Hospital (IRB number: 20141009). All subjects signed informed consent. A total of 129 patients with lung adenocarcinoma who consented to treatment at the Shanghai General Hospital between October 2014 and September 2016 were included in the study. Patients were enrolled based on the following inclusion criteria: 1) tissue samples were diagnosed by histopathological examination and patients signed informed consents before surgery, 2) no preoperative treatment, 3) normal functions of main organs (liver, heart, and kidney), 4) complete clinical records. A total of 129 pairs of cancer and adjacent non-cancerous tissues were obtained by surgical resection and stored in liquid nitrogen. The clinical characteristics of patients and five-year overall survival information were collected, recorded, and anonymized for analysis of clinical data.

### Online Database Validation

An online database starBase v2.0 [17] (https://starbase.sysu.edu.cn/index.php) was employed to validate the clinical results, including the expression pattern and prognostic value of CARD8-AS1.

### Cell Culture

The lung adenocarcinoma cell lines, including A549, NCI-H23, PC9, H1975, Calu-3, and normal lung cells, BEAS-1B, were purchased from the Cell Bank of Type Culture Collection of the Chinese Academy of Sciences (Shanghai, China). The DMEM (Gibco, MD, USA) supplemented with 10% FBS was used to culture cells in a humidified atmosphere with 5% CO_2_ were used for cell culture at 37°C.

### Cell Transfection

The pcDNA3.1-CARD8-AS1 (namely CARD8-AS1), and negative control pcDNA3.1 plasmid (namely NC) plasmids, miR-650 mimic (5′-AGG​AGG​CAG​CGC​UCU​CAG​GAC-3′), mimic negative control (mimic NC, 5′-GGA​CCA​AAT​CTC​GAG​ATT​TGG-3′), miR-650 inhibitor (5′-GUC​CUG​AGA​GCG​CUG​CCU​CCU-3′), and inhibitor NC (5′-UUC​UCC​GAA​CGU​GUC​ACG​UU-3′) were purchased from Shanghai GenePharma (China). The above plasmid constructs were transfected into A549 and PC9 cells using Lipofectamine 2000 (Invitrogen, Carlsbad, USA). The control group comprised untreated cells.

### Real-Time Quantitative PCR

Total RNA isolation from lung adenocarcinoma tissues was performed using TRIzol reagent (Invitrogen). The total RNA was reverse transcribed into cDNA using Primpscript RT reagent kit (TaKaRa, Dalian, China) or Mir-X miRNA First-Strand Synthesis Kit (TaKaRa) for lncRNA or miRNA, respectively, before RT-qPCR. Subsequently, RT-qPCR was conducted using the SYBR Premix Ex Taq kit (Takara) or Taqman miRNA assays (Applied Biosystems) in triplicates. The relative expression of CARD8-AS1 or miR-650 were normalized to that of GAPDH or U6, respectively. The sequences for PCR were as follows: CARD8-AS1, 5′–CCT​CCC​AGG​TTC​AAG​CGA​TT-3′ (forward) and 5′-GAT​TCC​TCC​AGG​CTG​TGA​CC-3′ (reverse); miR-650, 5′-GCC​GAG​AGG​AGG​CAG​CGC​T-3′ (forward) and 5′- CTC​AAC​TGG​TGT​CGT​GGA-3′ (reverse); GAPDH, 5′-CTG​GGC​TAC​ACT​GAG​CAC​C-3' (forward) and 5′-AAG​TGG​TCG​TTG​AGG​GCA​ATG-3′ (reverse); and U6, 5′-GCT​TCG​GCA​GCA​CAT​ATA​CTA​AAA​T-3′ (forward) and 5′-CGC​TTC​ACG​AAT​TTG​CGT​GTC​AT-3′ (reverse).

### CCK-8 Viability Assay

Cell proliferation was measured by a cell counting kit-8 assay. The cells (2000 cells) were seeded into 96-well plates after 24 h of transfection. The 10 μl CCK-8 (Dojindo, Japan) reagent was added to each well after culture for 0, 24, 48, and 72 h, respectively. After incubation for 2 h at 37°C, the optical density (OD) value at 450 nm was measured using a microplate reader.

### Western Blot Assay

The expression changes of Bcl-2 and Bax protein were determined using a western blot assay. After transfection, cells were collected and lysed with RIPA buffer to extract protein. The protein concentration was measured using the BCA protein assay kit (Beyotime). Then protein was separated using denaturing 10% SDS-PAGE and transferred to nitrocellulose membranes. The membranes were treated with blocking buffer and incubated with the primary antibodies against Bcl-2, Bax, or GAPDH (Abcam) at 4°C overnight, respectively. After washing with TBST, the membranes were incubated with the secondary antibody (Abcam) at 37°C for 1 h. Protein signals were detected using an ECL detection system and quantified using ImageJ software.

### Transwell Migration and Invasion Assays

For the transwell migration assay, the A549 and PC9 cells (4 × 10^4^ cells) in DMEM medium without FBS were placed at the top chamber (Corning, USA) with a noncoated membrane. To the lower chambers, a 500 μl DMEM medium with 10% FBS was added. After incubation for 24 h, the cells on the lower surface of the membrane were stained with 0.1% crystal violet for 30 min. For the transwell invasion assay, the matrigel chamber (BD, San Jose, USA) was used. The other steps were in line with those of the transwell migration assay. The cells were counted in five fields of view for each sample under a microscope.

### Luciferase Reporter Assay

The online database LncBase Predicted v.2 and Targetscan were used to assess the potential targets of lncRNA and miRNA, respectively. Among these miRNAs, miR-650 was reportedly upregulated in lung adenocarcinoma and had a prognostic value [18], thus it was chosen to confirm its interplay with CARD8-AS1.

The wild-type (WT-) or mutant (MUT-) constructs of CARD8-AS1 3′UTR, Bax 3′UTR containing miR-650 binding sites or a mutation at the predicted miR-650 was amplified, respectively, were amplified and fused with the pmirGLO (Promega, WI, USA) dual-luciferase vector. A549 and PC9 cells were co-transfected with miR-650 mimic, inhibitor, or NC and either CARD8-AS1 WT or MUT, as well as Bax WT or MUT, correspondingly. After 24 h of transfection, the firefly luciferase activity was detected using the dual-luciferase reporter kit (Promega, WI, USA) and normalized against the activity of Renilla luciferase activities.

### Statistical Analysis

All statistical analyses were conducted and diagrams were drawn using the SPSS 20.0 software (SPSS, Chicago, IL, USA) and GraphPad Prism version 7.0 (GraphPad Software, CA, USA). Data were presented as mean ± standard deviation of three or more independent experiments. The Kaplan-Meier curve and multiple Cox regression analyses were used to evaluate the clinical prognostic significance of CARD8-AS1. For comparison of the differences between two groups, paired Students’ t-test was used, and for comparison of differences among three or more groups, one-way ANOVA or two-way ANOVA was employed. The χ^2^ test was used to analyze the association between CARD8-AS1 expression and clinical characteristics of patients. The criterion of statistical significance was defined at a *p*-value less than 0.05.

## Results

### CARD8-AS1 Expression is Downregulated and is Inversely Associated With Overall Survival of Patients With Lung Adenocarcinoma

**FIGURE 1:**
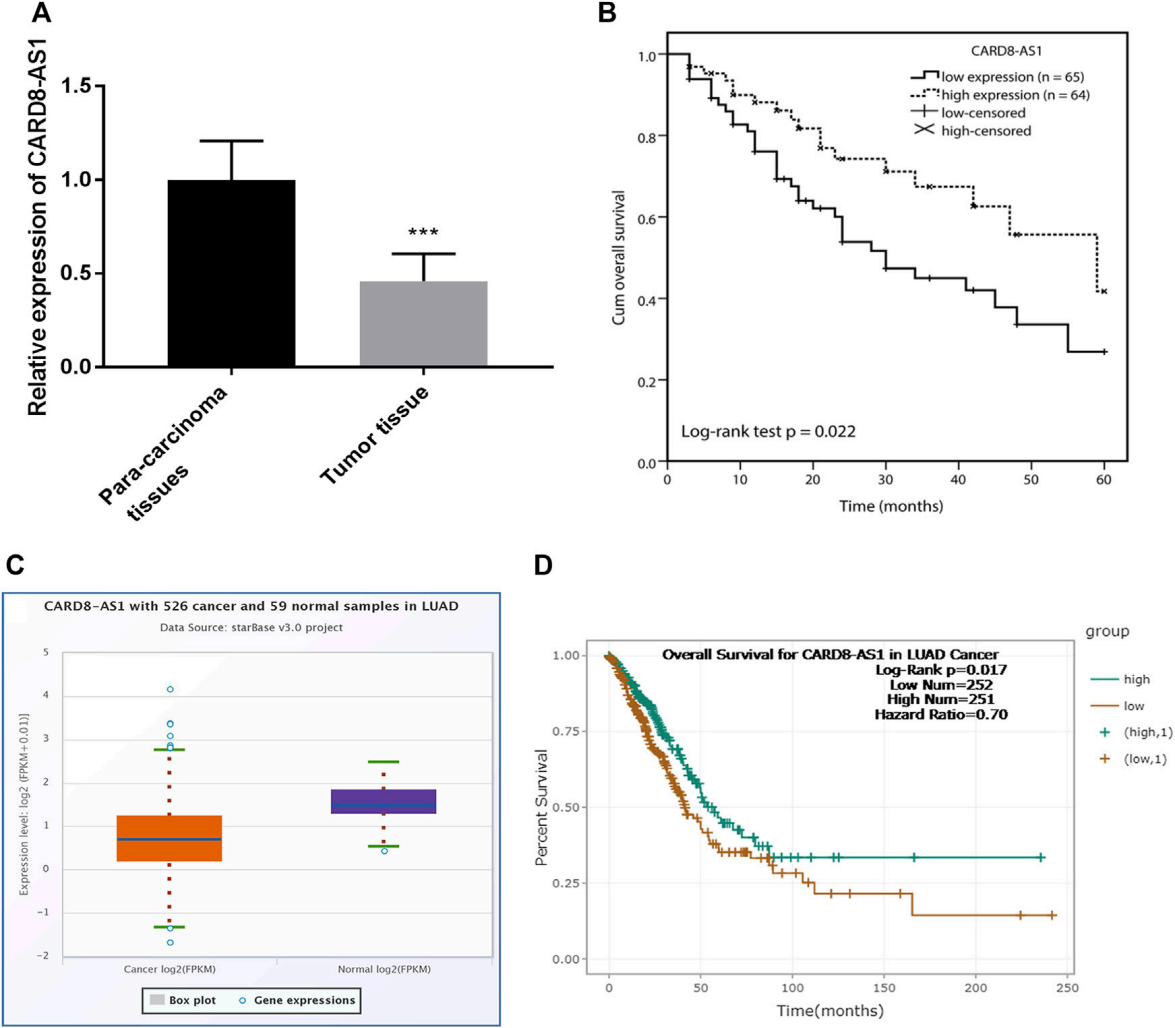
Expression and clinical prognostic role of CARD8-AS1 in lung adenocarcinoma. **(A)** CARD8-AS1 is downregulated in lung adenocarcinoma tissues as compared to para carcinoma normal tissues (***p < 0.001). **(B)** Kaplan-Meier analysis showed that low expression of CARD8-AS1 is associated with an adverse overall survival (log-rank test *p* = 0.022). **(C)** Validation of the expression of CARD8-AS1 using an online database starBase v3.0 from 526 cancer and 59 normal samples in lung adenocarcinoma. **(D)** Validation the prognostic value of CARD8-AS1 in lung adenocarcinoma (log-rank *p* = 0.017).

Initially, CARD8-AS1 expression in lung adenocarcinoma samples was assessed by RT-qPCR. The data in Figure 1A showed that CARD8-AS1 expression was down-regulated in lung adenocarcinoma tumor tissues as compared to para carcinoma normal tissues (*p* < 0.001). According to the median CARD8-AS1 expression level, all the patients were divided into the low CARD8-AS1- (*n* = 65) and the high CARD8-AS1 groups (*n* = 64). Subsequently, the clinical analyses revealed that low CARD8-AS1 expression was associated with tumor size (*p* = 0.042), TNM stage (*p* = 0.021), and lymph node metastasis (*p* = 0.025) but showed no significant relationship with age, sex, history of smoking or histological grade (Table 1).

**TABLE 1:** Association between CARD8-AS1 and clinicopathological characteristics in 129 patients with lung adenocarcinoma.

Variable	Cases	CARD8-AS1 expression		*p*-value
	*n* = 129	Low (*n* = 65)	High (*n* = 64)	
Age(years)				0.789
≤50	62	32	30	
>50	67	33	34	
Sex				0.181
Female	49	21	28	
Male	80	44	36	
Smoking				0.187
No	57	25	32	
Yes	72	40	32	
Tumor size(cm)				0.042
≤3	69	29	40	
>3	60	36	24	
Histological grade				0.127
Well and moderate	74	33	41	
Poor	55	32	23	
TNM stage				0.021
I/II	82	35	47	
III/IV	47	30	17	
Lymph node metastasis				0.025
No	74	31	43	
Yes	55	34	21	

Kaplan-Meier curve analysis with log-rank test showed that lung adenocarcinoma patients with low levels of CARD8-AS1 expression had poor survival compared to those with high CARD8-AS1 expression (log-rank test *p* = 0.022, Figure 1B). Further, multivariate Cox regression analysis suggested that CARD8-AS1 expression (*p* = 0.022) and TNM stage (*p* = 0.023) were independent risk factors associated with survival, which implied that CARD8-AS1 expression might be an independent prognostic risk factor associated with the overall survival of patients with lung adenocarcinoma (Supplementary Table S1).

### Verification of the Expression of CARD8-AS1 and Prognosis Using an Online Database

The expression of CARD8-AS1 and its prognostic value was confirmed using an online database starBase v2.0, in which the expression data of genes in cancers were downloaded from the TCGA project [17]. The expression of CARD8-AS1 in 526 cancer samples was lower compared with non-cancerous tissue samples (Figure 1C). Moreover, patients with low CARD8-AS1 expression exhibited a poor survival rate compared to patients with high CARD8-AS1 (log-rank *p* = 0.017, Figure 1D). The above data confirmed the downregulation of CARD8-AS1 and its prognostic value in lung adenocarcinoma.

### Downregulation of CARD8-AS1 Weakens the Cellular Behaviors of A549 and PC9 Cells

**FIGURE 2:**
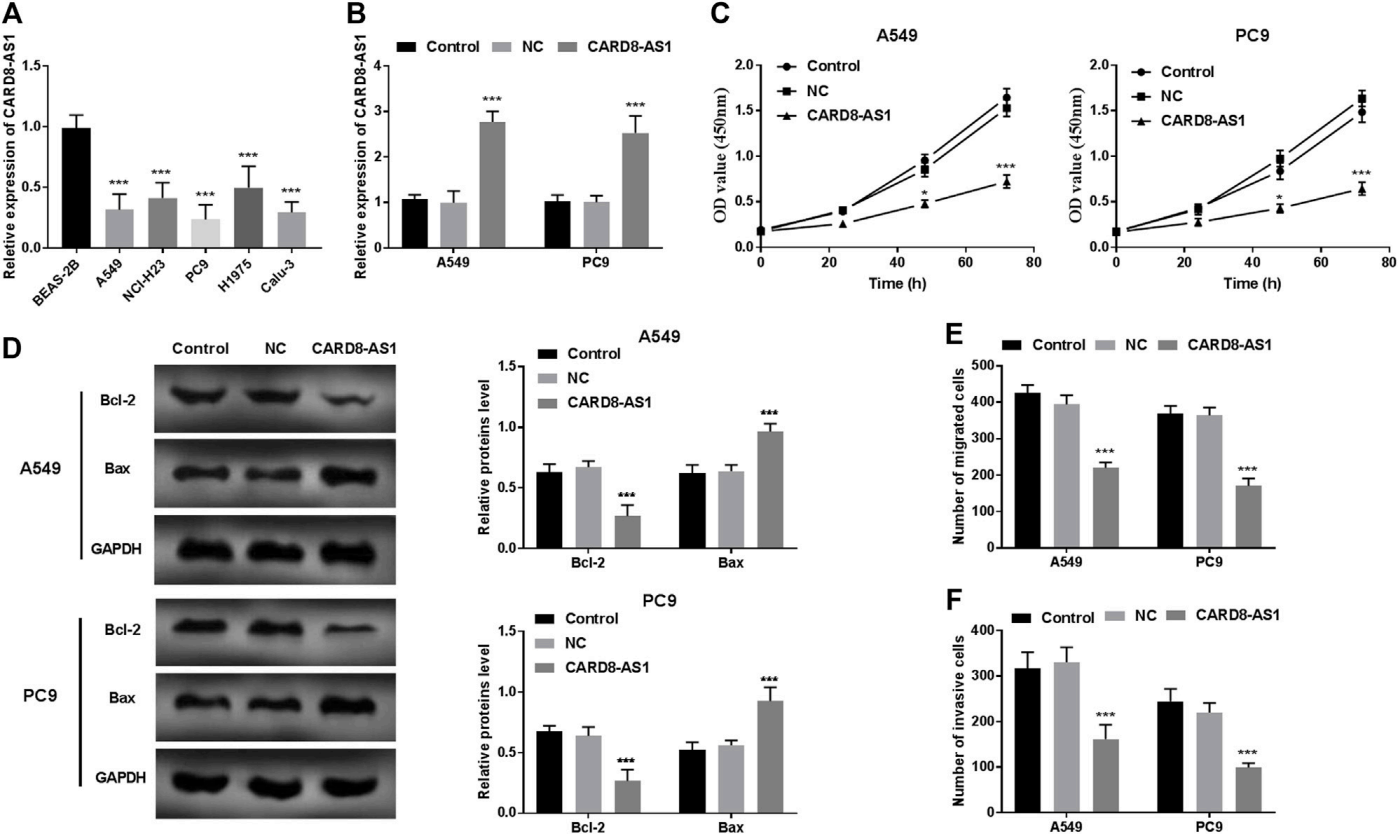
Functional role of CARD8-AS1 in A549 and PC9 cells. **(A)** Expression of CARD8-AS1 is lower in lung adenocarcinoma cell lines (A549, NCI-H23, PC9, H1975, and Calu-3) as compared to that in the normal lung cell line, BEAS-2B (***p < 0.001). **(B)** Cell transfection was conducted in A549 and PC9 cells using pcDNA3.1-CARD8-AS1 (***p < 0.001) plasmid construct. **(C)** Overexpression of CARD8-AS1 suppresses proliferation of A549 and PC9 cells (*p < 0.05, ***p < 0.001). **(D)** The Bcl-2 and Bax protein levels were measured using a western blot assay (***p < 0.001). **(E)** Increased expression of CARD8-AS1 restrains cellular migration abilities (***p < 0.001). **(F)** The invasive capacities of lung adenocarcinoma cells are inhibited by CARD8-AS1 overexpression (***p < 0.001).

CARD8-AS1 expression in lung adenocarcinoma cells was evaluated to determine its potential role in the progression of lung adenocarcinoma. The expression of CARD8-AS1 was decreased significantly in the lung adenocarcinoma cells (A549, NCI-H23, PC9, H1975, and Calu-3) as compared to the normal BEAS-2B cells (*p* < 0.001, Figure 2A).

To elucidate the role of CARD8-AS1 in lung adenocarcinoma, the pcDNA3.1-CARD8-AS1 plasmid was transfected into A549 and PC9 cells and the results as shown in Figure 2B suggested that CARD8-AS1 expression increased dramatically in transfected cells with pcDNA3.1-CARD8-AS1 (*p* < 0.001). The results of the CCK-8 assay revealed that the proliferation of A549 and PC9 cells reduced significantly upon transfection with CARD8-AS1 (*p* < 0.05, Figure 2C). Moreover, apoptotic molecules, Bcl-2 and Bax, were measured using a western blot assay. We observed that overexpression of CARD8-AS1 decreased Bcl-2 protein levels and increased Bax protein levels (*p* < 0.001, Figure 2D). Consistent with proliferation assay results, the migration ability (Figure 2E) and invasive capacity (Figure 2F) of A549 and PC9 cells were inhibited by CARD8-AS1 upregulation (*p* < 0.001).

### CARD8-AS1 Sponges miR-650 in A549 Lung Adenocarcinoma Cells

**FIGURE 3:**
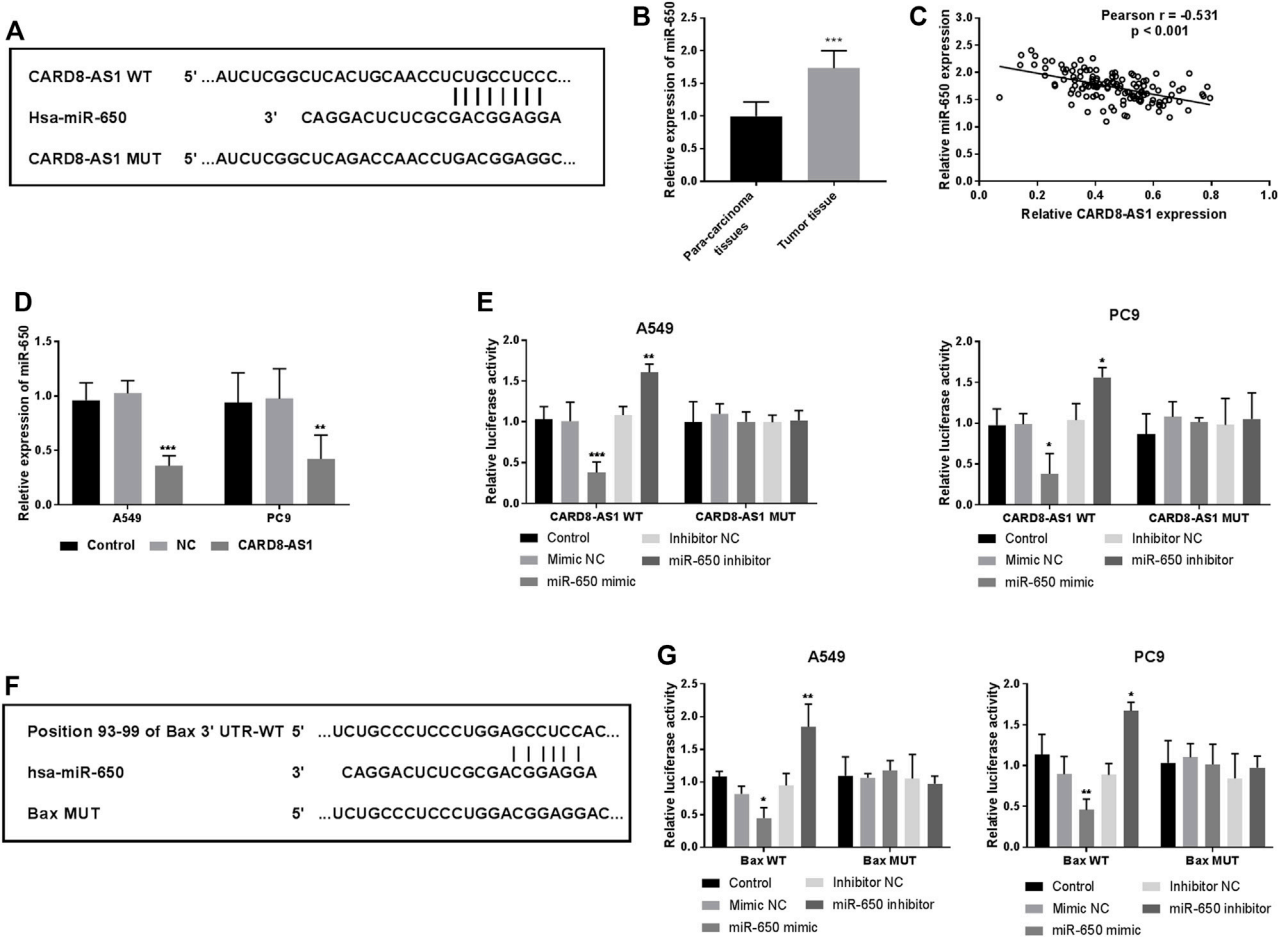
miR-650 is a potential target of CARD8-AS1, and Bax is a direct target of miR-650. **(A)** The putative binding sites for CARD8-AS1 and miR-650. **(B)** miR-650 expression increases in tumor tissues as compared to para-carcinoma tissues (***p < 0.001). **(C)** Correlation analysis between CARD8-AS1 and miR-650 expressions by Pearson analysis in lung adenocarcinoma tissues (r = −0.531, *p* < 0.001). **(D)** The expression of miR-650 decreases upon CARD8-AS1 overexpression in A549 cells (***p < 0.001). **(E)** Luciferase activity was measured in A549 and PC9 cells co-transfected with WT-CARD8-AS1 or MUT-CARD8-AS1 and miR-650 mimic, inhibitor, or NCs (*p < 0.05, **p < 0.01, ***p < 0.001). **(F)** The binding sites between Bax and miR-650. **(G)** Luciferase activity was measured in A549 and PC9 cells co-transfected with WT-Bax or MUT-Bax and miR-650 mimic, inhibitor, or NCs. (*p < 0.05, **p < 0.01).

We hypothesized that CARD8-AS1 upregulation restrained the tumorigenesis and progression of lung adenocarcinoma cells. The potential CARD8-AS1-related miRNAs were subsequently examined. As predicted by the bioinformatics database, Tool LncBase Predicted v.2, CARD8-AS1 contained binding sites which were complementary bound to the “seed sequence” of miR-650, as illustrated in Figure 3A. The expression of miR-650 was evaluated in tissue samples and the data suggested its upregulation in tumor tissues as compared to the para-carcinoma normal tissues (*p* < 0.001, Figure 3B). Moreover, the relative expressions of CARD8-AS1 and miR-650 were inversely correlated (*p* < 0.001, Figure 3C), whereby overexpression of CARD8-AS1 decreased miR-650 expression (*p* < 0.001, Figure 3D). To verify the interplay between CARD8-AS1 and miR-650, a dual-luciferase reporter assay was performed. The data showed that the relative luciferase activity of a vector expressing CARD8-AS1 WT decreased upon cotransfection with miR-650 mimic whilst being elevated with cotransfection with miR-650 inhibitor (*p* < 0.01) rather than CARD8-AS1 MUT (Figure 3E). These data confirmed that CARD8-AS1 could interact with miR-650 and served as a sponge for miR-650.

### Bax May Be a Potential Target of miR-650

Using the Targetscan online database, the binding sites between miR-650 and Bax were observed and are shown in Figure 3F. The dual-luciferase reporter assay indicated that the relative luciferase activities of Bax WT cells were decreased by a miR-650 mimic while increased by a miR-650 inhibitor (*p* < 0.05, Figure 3G). Whereas, the luciferase activity has no change in Bax MUT cells (Figure 3G). These findings suggested that Bax may be a direct target of miR-650.

## Discussion

In this study, lncRNA CARD8-AS1 was found to be downregulated in lung adenocarcinoma tissues and cells and could potentially mediate miR-650 function in lung adenocarcinoma. The downregulation of CARD8-AS1 was significantly associated with tumor size, TNM stage, lymph node metastasis, and poor survival. In cell lines increased expression of CARD8-AS1 reduces cell viability and affects cell migration and invasion abilities in lung adenocarcinoma cells by targeting miR-650.

Accumulating evidence demonstrates the functional and clinical roles of lncRNAs involved in tumor progression [19–21]. For instance, lincRNA OIN1 is oncogenic in ovarian cancer and a potential molecular target for its treatment [22]. Higher lncRNA ANRIL expression is related to increased metastases rates and reduced overall survival rate in osteosarcoma [23]. These studies suggest the vital roles of lncRNAs in regulating tumor progressions. In this study, CARD8-AS1 was downregulated in lung adenocarcinoma tissues, which suggested its role as a tumor suppressor. Moreover, the expression of CARD8-AS1 was related to clinical characteristics, such as TNM stage, lymph node metastasis, and tumor size, which implied that CARD8-AS1 expression may be associated with tumor progression.

Previous studies indicate the potential value of lncRNAs in the prognosis of several tumors [24, 25]. CARD8-AS1 is an aberrantly expressed lncRNA and is closely associated with ovarian cancer patients’ overall survival [14]. In the present study, the findings show that low CARD8-AS1 expression is associated with lung adenocarcinoma patients’ five-year overall survival. Additionally, CARD8-AS1 expression was an independent prognostic risk factor. These results suggested that CARD8-AS1 may serve as a prognostic biomarker in lung adenocarcinoma.

lncRNAs can regulate tumor progression through altering cellular viability, cellular migration, and invasion [26, 27]. To evaluate the functional role of CARD8-AS in lung adenocarcinoma, its expression in different lung adenocarcinoma cell lines was assessed. In line with the expression patterns in tumor tissues, CARD8-AS1 expression was also decreased in lung adenocarcinoma cell lines. Further results of the cellular experiments revealed that elevated expression of CARD8-AS1 reduced A549 and PC9 cell proliferation capacities, migration, and invasion potential. Moreover, upregulation of CARD8-AS1 decreased Bcl-2 protein levels and increased Bax protein levels. In all, these data implied that CARD8-AS1 may play tumor-inhibitory roles in the progression of lung adenocarcinoma.

The lncRNAs can target several miRNAs and the lncRNA-miRNA network serves a crucial role in tumors [28, 29]. For instance, linc00337 expression increases in lung adenocarcinoma, while its knockdown in cell lines could suppress cellular activities by targeting miR-1285-3p [27]. In this study, the potential CARD8-AS1-related miRNAs were predicted and examined. Among them, miR-650 was chosen to verify its interplay with CARD8-AS1. Several previous studies demonstrated that miR-650 expression is upregulated in lung cancer and can promote tumor cell proliferation and invasion, as well as be a prognostic factor for lung adenocarcinoma [18, 30, 31]. The results of this study suggest that miR-650 is a target miRNA of CARD8-AS1 and that CARD8-AS1 may act as a miR-650 sponge in lung adenocarcinoma. miR-650 is upregulated in many tumor tissues and plays oncogenic roles in these types of cancer [32–35]. In lung cancer, miR-650 could promote cellular proliferation and invasion, as well as confer the chemoresistance to docetaxel by regulating Bcl-2/Bax expression [18, 30]. Furthermore, we observed that Bax may be a direct target of miR-650. Thus, we hypothesized that CARD8-AS1 may suppress lung adenocarcinoma cell proliferation, migration, and invasion by targeting miR-650 and then regulating Bax expression.

## Conclusion

In conclusion, LncRNA CARD8-AS1 was downregulated and related to several crucial clinical parameters, and consequently may serve as a prognosis marker in lung adenocarcinoma. In cell lines increased CARD8-AS1 suppressed tumor cell proliferation, migration, and invasion by regulating miR-650/Bax. Thus, CARD8-AS1 may inhibit tumor progression in lung adenocarcinoma by targeting the miR-650/Bax axis, which underlies its value and utility as a prognostic marker for lung adenocarcinoma patients.

Our data represents an advance in biomedical science as it provides a framework for the potential of CARD8-AS1 as a tumor-suppressor in the progression of lung adenocarcinoma.

## Limitations

Some limitations of the current study will be addressed in our future studies. Firstly, the clinical samples were from a single center and the sample size was small, and it needs a larger sample size to confirm the potential clinical significance of CARD8-AS1 in lung adenocarcinoma. Another limitation is that the present functional study was carried out using cell lines *in vitro*. Thus, the detailed mechanism of CARD8-AS1 in lung adenocarcinoma will be further investigated *in vivo* using an animal model.

## Summary Table

### What Is Known About This Subject

• lncRNA CARD8-AS1 shows reduced expression in several cancers.

### What This Study Adds

• Low CARD8-AS1 levels are related to tumor size, lymph node metastasis, TNM stage, and poor overall survival.
• CARD8-AS1 may play a tumor-suppressive role in lung adenocarcinoma by regulating miR-650/Bax axis.

## Data Availability

The original contributions presented in the study are included in the article/Supplementary Material, further inquiries can be directed to the corresponding authors.

## References

[1] Siegel RL Miller KD Fuchs HE Jemal A. Cancer Statistics, 2021. CA Cancer J Clin (2021) 71:7–33. 10.3322/caac.21654 33433946

[2] Ruiz-Cordero R Devine WP. Targeted Therapy and Checkpoint Immunotherapy in Lung Cancer. Surg Pathol Clin (2020) 13:17–33. 10.1016/j.path.2019.11.002 32005431

[3] Hirsch FR Scagliotti GV Mulshine JL Kwon R Curran WJ Wu Y-L Lung Cancer: Current Therapies and New Targeted Treatments. The Lancet (2017) 389:299–311. 10.1016/s0140-6736(16)30958-8 27574741

[4] Yang S Zhang Z Wang Q. Emerging Therapies for Small Cell Lung Cancer. J Hematol Oncol (2019) 12:47. 10.1186/s13045-019-0736-3 31046803 PMC6498593

[5] Zeng X Xu C Cheng J Sun C Wang Z Gong Z Poor Glycemic Control Might Compromise the Efficacy of Chemotherapy in Non‐small Cell Lung Cancer Patients with Diabetes Mellitus. Cancer Med (2020) 9:902–11. 10.1002/cam4.2750 31830375 PMC6997083

[6] Jarroux J Morillon A Pinskaya M. History, Discovery, and Classification of lncRNAs. Adv Exp Med Biol (2017) 1008:1–46. 10.1007/978-981-10-5203-3_1 28815535

[7] Marchese FP Raimondi I Huarte M. The Multidimensional Mechanisms of Long Noncoding RNA Function. Genome Biol (2017) 18:206. 10.1186/s13059-017-1348-2 29084573 PMC5663108

[8] Han X Jiang H Qi J Li J Yang J Tian Y Novel lncRNA UPLA1 Mediates Tumorigenesis and Prognosis in Lung Adenocarcinoma. Cell Death Dis (2020) 11:999. 10.1038/s41419-020-03198-y 33221813 PMC7680460

[9] Zhao M Xin XF Zhang JY Dai W Lv TF Song Y. LncRNA GMDS‐AS1 Inhibits Lung Adenocarcinoma Development by Regulating miR‐96‐5p/CYLD Signaling. Cancer Med (2020) 9:1196–208. 10.1002/cam4.2776 31860169 PMC6997056

[10] Peng W-X Koirala P Mo Y-Y. LncRNA-mediated Regulation of Cell Signaling in Cancer. Oncogene (2017) 36:5661–7. 10.1038/onc.2017.184 28604750 PMC6450570

[11] Zheng S Yang L Zou Y Liang J-y. Liu P Gao G Long Non-coding RNA HUMT Hypomethylation Promotes Lymphangiogenesis and Metastasis via Activating FOXK1 Transcription in Triple-Negative Breast Cancer. J Hematol Oncol (2020) 13:17. 10.1186/s13045-020-00852-y 32138762 PMC7059688

[12] Wang N Yu Y Xu B Zhang M Li Q Miao L. Pivotal Prognostic and Diagnostic Role of the Long Non-coding RNA colon Cancer-Associated Transcript 1 Expression in Human Cancer (Review). Mol Med Rep (2019) 19:771–82. 10.3892/mmr.2018.9721 30535444 PMC6323215

[13] Fan G Jiao J Shen F Chu F. Upregulation of lncRNA ZFAS1 Promotes Lung Adenocarcinoma Progression by Sponging miR ‐1271‐5p and Upregulating FRS2. Thorac Cancer (2020) 11:2178–87. 10.1111/1759-7714.13525 32515146 PMC7396366

[14] Li N Zhan X. Identification of Clinical Trait-Related lncRNA and mRNA Biomarkers with Weighted Gene Co-expression Network Analysis as Useful Tool for Personalized Medicine in Ovarian Cancer. EPMA J (2019) 10:273–90. 10.1007/s13167-019-00175-0 31462944 PMC6695468

[15] Lin X Jiang T Bai J Li J Wang T Xiao J Characterization of Transcriptome Transition Associates Long Noncoding RNAs with Glioma Progression. Mol Ther Nucleic Acids (2018) 13:620–32. 10.1016/j.omtn.2018.10.009 30472640 PMC6251785

[16] Zhao T Khadka VS Deng Y. Identification of lncRNA Biomarkers for Lung Cancer through Integrative Cross-Platform Data Analyses. Aging (2020) 12:14506–27. 10.18632/aging.103496 32675385 PMC7425463

[17] Li J-H Liu S Zhou H Qu L-H Yang J-H. starBase v2.0: Decoding miRNA-ceRNA, miRNA-ncRNA and Protein-RNA Interaction Networks from Large-Scale CLIP-Seq Data. Nucl Acids Res (2014) 42:D92–D97. 10.1093/nar/gkt1248 24297251 PMC3964941

[18] Huang J-Y Cui S-Y Chen Y-T Song H-Z Huang G-C Feng B MicroRNA-650 Was a Prognostic Factor in Human Lung Adenocarcinoma and Confers the Docetaxel Chemoresistance of Lung Adenocarcinoma Cells via Regulating Bcl-2/Bax Expression. PLoS One (2013) 8:e72615. 10.1371/journal.pone.0072615 23991130 PMC3749147

[19] Jiang J Lu Y Zhang F Huang J Ren X-l. Zhang R. The Emerging Roles of Long Noncoding RNAs as Hallmarks of Lung Cancer. Front Oncol (2021) 11:761582. 10.3389/fonc.2021.761582 34692550 PMC8529012

[20] Peng N Zhang Z Wang Y Yang M Fan J Wang Q Down-regulated LINC00115 Inhibits Prostate Cancer Cell Proliferation and Invasion via Targeting miR-212-5p/FZD5/Wnt/β-Catenin axis. J Cel Mol Med (2021). 25:10627. 10.1111/jcmm.17000 PMC8581327 34697900

[21] Zhao X Wu J Li Y Ye F Wang C. Long Non-coding RNA FENDRR Inhibits the Stemenss of Colorectal Cancer Cells through Directly Binding to Sox2 RNA. Bioengineered (2021) 12:8698–708. 10.1080/21655979.2021.1977054 34697986 PMC8806690

[22] Takeiwa T Mitobe Y Ikeda K Hasegawa K Horie K Inoue S. Long Intergenic Noncoding RNA OIN1 Promotes Ovarian Cancer Growth by Modulating Apoptosis-Related Gene Expression. Int J Mol Sci (2021) 22:11242. 10.3390/ijms222011242 34681900 PMC8541687

[23] Lee AM Ferdjallah A Moore E Kim DC. Long Non-coding RNA ANRIL as a Potential Biomarker of Chemosensitivity and Clinical Outcomes in Osteosarcoma. Int J Mol Sci (2021) 22:11168. 10.3390/ijms222011168 34681828 PMC8538287

[24] Ebrahimi AA Ashoori H Vahidian F Mosleh IS Kamian S. Long Non-coding RNA Panel as a Molecular Biomarker in Glioma. J Egypt Natl Canc Inst (2021) 33:31. 10.1186/s43046-021-00090-4 34693506 PMC13316911

[25] Li Z Zhuo Y Li J Zhang M Wang R Lin L. Long Non-coding RNA SNHG4 is a Potential Diagnostic and Prognostic Indicator in Non-small Cell Lung Cancer. J Egypt Natl Canc Inst (2021) 51:654–62. 34686507

[26] Tong S Wang X Guo X Lu Z. Knockdown of lncRNA IGF2BP2-AS1 Inhibits Proliferation and Migration of Oral Squamous Cell Carcinoma Cells via the Wnt/β-Catenin Pathway. J Oral Pathol Med (2021) 51:272. 10.1111/jop.13248 34637162

[27] Zhang R-n. Wu D-m. Wu L-p. Gao G-w. LncRNA LINC00337 Sponges Mir-1285-3p to Promote Proliferation and Metastasis of Lung Adenocarcinoma Cells by Upregulating YTHDF1. Cancer Cel Int (2021) 21:550. 10.1186/s12935-021-02253-8 PMC8524958 34663343

[28] Dai W Chao X Jiang Z Zhong G. lncRNA KCNQ1OT1 May Function as a Competitive Endogenous RNA in Atrial Fibrillation by Sponging miR-223-3p. Mol Med Rep (2021) 24:870. 10.3892/mmr.2021.12510 34698362 PMC8569515

[29] Vimalraj S Subramanian R Dhanasekaran A. LncRNA MALAT1 Promotes Tumor Angiogenesis by Regulating MicroRNA-150-5p/VEGFA Signaling in Osteosarcoma: *In-Vitro* and *In-Vivo* Analyses. Front Oncol (2021) 11:742789. 10.3389/fonc.2021.742789 34692524 PMC8529043

[30] Tang X Ding Y Wang X Wang X Zhao L Bi H. miR-650 Promotes Non-small Cell Lung Cancer Cell Proliferation and Invasion by Targeting ING4 through Wnt-1/β-Catenin Pathway. Oncol Lett (2019) 18:4621–8. 10.3892/ol.2019.10805 31611970 PMC6781663

[31] Zhao Y Zhu Z Shi S Wang J Li N. Long Non-coding RNA MEG3 Regulates Migration and Invasion of Lung Cancer Stem Cells via miR-650/SLC34A2 axis. Biomed Pharmacother (2019) 120:109457. 10.1016/j.biopha.2019.109457 31585300

[32] Farooqi AA Qureshi MZ Coskunpinar E Naqvi SK-U -H Yaylim I Ismail M. MiR-421, miR-155 and miR-650: Emerging Trends of Regulation of Cancer and Apoptosis. Asian Pac J Cancer Prev (2014) 15:1909–12. 10.7314/apjcp.2014.15.5.1909 24716910

[33] Han LL Yin XR Zhang SQ. miR-650 Promotes the Metastasis and Epithelial-Mesenchymal Transition of Hepatocellular Carcinoma by Directly Inhibiting LATS2 Expression. Cell Physiol Biochem (2018) 51:1179–92. 10.1159/000495495 30481780

[34] Orlandella FM Mariniello RM Iervolino PLC Imperlini E Mandola A Verde A miR-650 Promotes Motility of Anaplastic Thyroid Cancer Cells by Targeting PPP2CA. Endocrine (2019) 65:582–94. 10.1007/s12020-019-01910-3 30927143

[35] Zuo Z-H Yu YP Ding Y Liu S Martin A Tseng G Oncogenic Activity of miR-650 in Prostate Cancer is Mediated by Suppression of CSR1 Expression. Am J Pathol (2015) 185:1991–9. 10.1016/j.ajpath.2015.03.015 25956032 PMC4484220

