# Supplementary material for: Identification of LncRNA CARD8-AS1 as a Potential Prognostic Biomarker Associated With Progression of Lung Adenocarcinoma
Source: Br J Biomed Sci. 2022 Jun 23;79:10498. doi: 10.3389/bjbs.2022.10498 (PMC9302548; doi:10.3389/bjbs.2022.10498)
Supplement: Supplementary file 1 [file Table1.DOCX]

**Supplementary Table 1** Multivariate Cox analysis was conducted to predict the risk factors associated with overall survival.

| Variables | HR | 95%CI | *p*-value |
| --- | --- | --- | --- |
| CARD8-AS1 | 2.157 | 1.120-4.154 | 0.022 |
| Age | 1.646 | 0.903-2.998 | 0.104 |
| Sex | 1.686 | 0.928-3.066 | 0.087 |
| Smoking history | 1.709 | 0.924-3.163 | 0.088 |
| Tumor size | 1.361 | 0.748-2.477 | 0.313 |
| Differentiation | 1.836 | 0.972-3.468 | 0.061 |
| TNM stage | 1.942 | 1.094-3.449 | 0.023 |
| Lymph node metastasis | 1.749 | 0.983-3.110 | 0.057 |
